# L1 Syndrome Prenatal Diagnosis Supplemented by Functional Analysis of One *L1CAM* Gene Missense Variant

**DOI:** 10.1007/s43032-021-00828-4

**Published:** 2021-12-16

**Authors:** Ping Wang, Hong Liao, Quyou Wang, Hanbing Xie, He Wang, Mei Yang, Shanling Liu

**Affiliations:** 1 Department of Obstetrics & Gynecology, West China Second University Hospital, Sichuan University, No. 20, Section 3, Renminnan Road, Chengdu, 610041 Sichuan China; 2 Key Laboratory of Birth Defects and Related Diseases of Women and Children, Ministry of Education, Sichuan University, Chengdu, Sichuan China

**Keywords:** L1 syndrome, L1 cell adhesion molecule, Prenatal diagnosis, Functional analysis

## Abstract

L1 syndrome, a complex X-linked neurological disorder, is caused by mutations in the L1 cell adhesion molecule (*L1CAM*) gene. *L1CAM* molecule is a member of immunoglobulin (Ig) superfamily of neural cell adhesion molecules (CAMs), which plays a pivotal role in the developing nervous system. In this study, a *L1CAM* gene exonic missense variant (c.1108G > A, p.G370R) was identified in two induced fetuses (abnormal fetuses), who presented corpus callosum agenesis accompanied with hydrocephalus. Clinical data, published literature, online database, and bioinformatic analysis suggest that the single-nucleotide variant of *L1CAM* gene is a likely pathogenic mutation. In vitro assays were performed to evaluate the effects of this variant. Based on NSC-34/COS-7 cells transfected with wild-type (L1-WT) and mutated (L1-G370R) plasmids, the *L1CAM* gene exonic missense variant (c.1108G > A, p.G370R) reduced cell surface expression, induced partial endoplasmic reticulum retention, affected posttranslational modification, and reduced protein’s homophilic adhesive ability, but did not induce endoplasmic reticulum stress, which might probably associate with L1 syndrome. Finally, 35 isolated fetuses were screened for *L1CAM* gene variants by Sanger sequencing. These cases all prenatally suspected of corpus callosum agenesis accompanied with hydrocephalus, which may relate to L1 syndrome. Consequently, one *L1CAM* gene single missense variant (c.550C > T, p.R184W) was detected in one fetus. Our results provided evidence that the *L1CAM* gene missense variant (c.1108G > A, p.G370R) may relate to L1 syndrome. The findings of this study suggest a potential possibility of *L1CAM* gene screening for prenatal diagnoses for fetuses presented corpus callosum agenesis accompanied with hydrocephalus.

**Supplementary Information:**

The online version contains supplementary material available at 10.1007/s43032-021-00828-4.

## Introduction

L1 syndrome, a complex X-linked neurological disorder with 1:30,000 incidence in newborn males, is caused by mutations in the L1 cell adhesion molecule (*L1CAM*) gene [1, 2]. It comprises X-linked hydrocephalus due to stenosis of the aqueduct of Sylvius (HSAS; MIM #307,000), X-linked complicated corpus callosum agenesis (MIM #304,100), MASA syndrome (mental retardation, aphasia, shuffling gait, adducted thumbs; MIM #303,350), and X-linked complicated hereditary spastic paraplegia type 1 (MIM #303,350) [3].

The *L1CAM* gene consists of 29 exons, located in the Xq28 region, and encodes a neuronal cell adhesion molecule. *L1CAM* molecule is a member of the immunoglobulin (Ig) superfamily of neural cell adhesion molecules (CAMs), which plays pivotal roles in neurite outgrowth, pathfinding, and fasciculation, as well as neuronal cell migration and survival [4–11]. All L1-related molecules share a primary structural organization of six Ig-like motifs, followed by five fibronectin type III (FNIII)-like repeats at the extracellular surface, a hydrophobic transmembrane region, and a short cytoplasmic segment in the C-terminus [12–14]. In addition, the extracellular part of *L1CAM* protein is responsible for mediating homophilic interactions with the *L1CAM* protein itself and heterophilic interactions with several other cell adhesion molecules, extracellular matrix, and chondroitin sulfate proteoglycan [15–18].

At present, more than 270 different *L1CAM* gene mutations have been reported, and almost one-third of them are single missense mutations. Most of these missense variants are located in extracellular domains of *L1CAM* molecule, which generally result in a more severe phenotype than those affecting the cytoplasmic protein domain [19]. In addition, previous studies have suggested that such missense mutations may reduce cell surface expression and affect protein misfolding, homo- and heterophilic ligand binding, or intracellular processing. Finally, they might interfere with neurite growth and neurite branching.

Here, we reported one variant of *L1CAM* gene in two induced fetuses (abnormal fetuses) suspected of L1 syndrome, which is likely disease-causing. The study provided case presentation, protein functional analysis, and screening of *L1CAM* gene variants in isolated fetuses. Together, a prenatal diagnosis may be provided for fetuses presented corpus callosum agenesis accompanied with hydrocephalus.

### Case Presentation

#### Clinical Summary

**Fig. 1:**
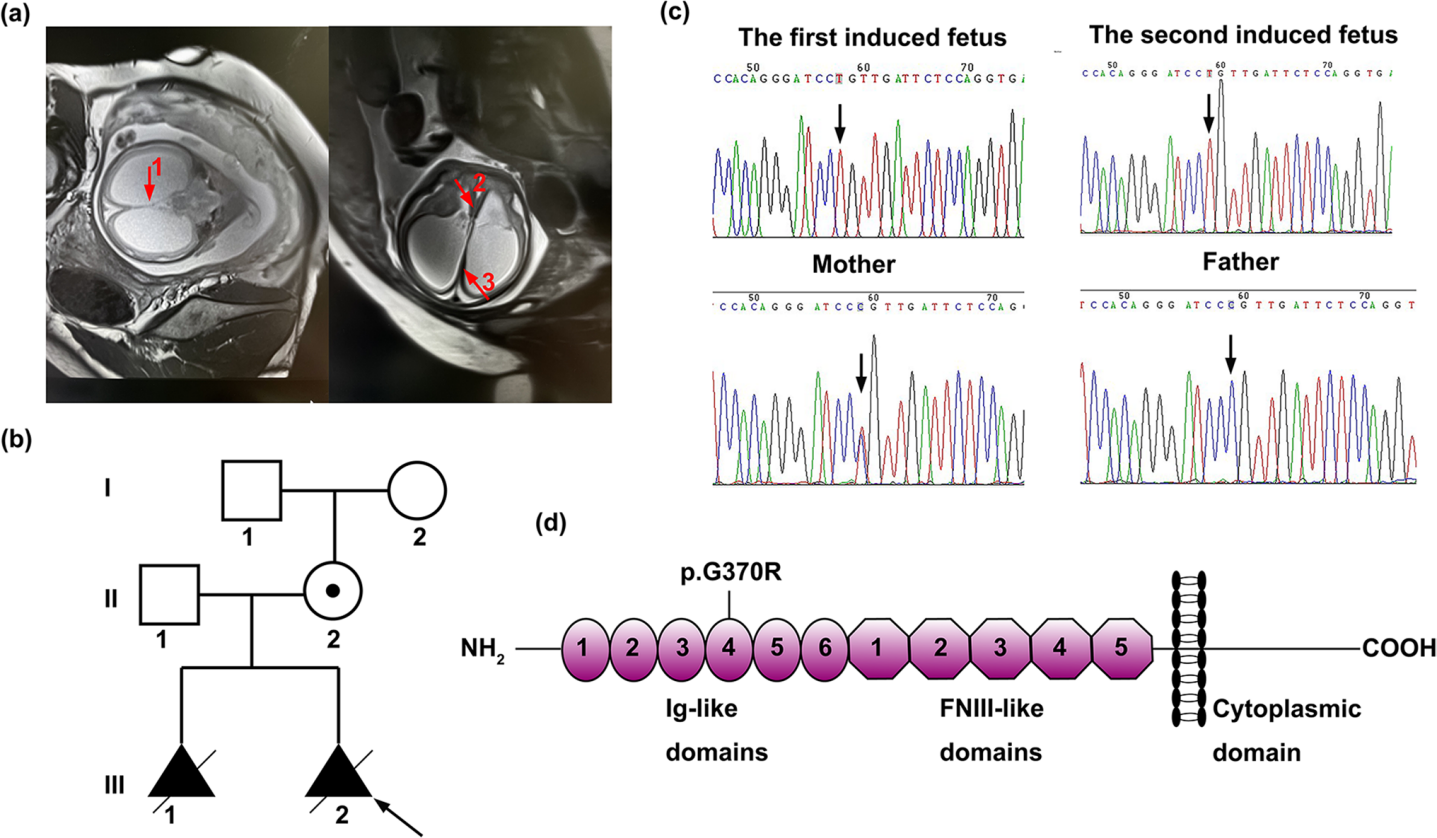
Clinical characteristics of family and schematic model of *L1CAM* molecule. **a** Magnetic resonance imaging of the second induced fetus (abnormal fetus). The absence of the cavum septi pellucidi is indicated by arrow 1 in the coronal plane. The corpus callosum agenesis is indicated by arrows 2 and 3 in the axial plane. **b** Pedigrees of the analyzed family in this study. Males are represented by squares, females are represented by circles, and triangle refers to abortion in early pregnancy. The proband is indicated by an arrow. **c** Sanger sequences of the *L1CAM* gene mutation (c.1108G > A). Hemizygous mutation detected in two induced fetuses (abnormal fetuses). The heterozygous mutation detected in these fetuses’ mother but not in these fetuses’ father. Mutation was indicated by arrow. **d** Schematic model of *L1CAM* molecule bearing domain structure: six immunoglobulin-like motifs (Ig-like), five fibronectin type III (FNIII)-like domains, a transmembrane region, and an intracellular domain (ICD). The location of the amino acid substitution (p.G370R) is indicated

A 25-year-old, G2P0^+2^ woman (II.2) came to the genetic counseling clinic of West China Second University Hospital, Sichuan University (Chengdu, China). The woman informed the doctor that her first fetus presented corpus callosum agenesis through an ultrasound examination during the second trimester. Then, she required a termination of pregnancy. Her second fetus presented corpus callosum agenesis accompanied with hydrocephalus, narrowness of brain parenchyma, and the absence of the cavum septi pellucidi (CSP), from the reports of fetal ultrasound scan and magnetic resonance imaging (Fig. 1a). The woman was seen for genetic examination of the second induced fetus (abnormal fetus) and genetic counseling for the next pregnancy. The parents and maternal grandparents of the proband had no related clinical manifestations. The pedigree of this family is shown in Fig. 1b.

#### Genetic Findings

DNA extracted from the muscle of the second induced fetus (abnormal fetus) and peripheral blood of parents was tested by trio whole-exome sequencing (Trio-WES). Candidate variant was validated using Sanger sequencing. Polymerase chain reaction (PCR) amplification was performed using primers (Supplementary Table 1) designed to cover variant identified by WES. In summary, exome capture sequencing was performed using Nano WES Human Exome V1 (Berry genomics) and Illunima NovaSeq6000 platform with 150-bp paired-end reads. Burrows-Wheeler Aligner software tool was used for aligning the sequencing reads with hg38/GRCh38. Local alignment and recalibration of the base quality of the Burrows-Wheeler aligned reads was performed by the GATK Indel Realigner and the GATK Base Recalibrator, respectively (broadinstitute.org/). Then, single-nucleotide variants (SNVs) and small insertions or deletions (InDels) were identified by GATK Unified Genotyper (broadinstitute.org). Variants were annotated and interpreted using ANNOVAR and the Enliven Variants Annotation Interpretation System (Berry genomics). Public databases used for filtering include gnomAD (http://gnomad.broadinstitute.org/), 1000 Genomes Project (1000G) (http://browser.1000genomes.org), etc. Pathogenicity of SNVs was evaluated based on the scientific medical literature and disease databases, such as PubMed (https://www.ncbi.nlm.nih.gov/pubmed/), ClinVar (http://www.ncbi.nlm.nih.gov/clinvar), OMIM (http://www.omim.org), Human Gene Mutation Database (http://www.hgmd.org), and Human Genome Variation Society (http://www.hgvs.org/dblist/dblist.html).

A hemizygous variant (c.1108G > A, p.G370R) in *L1CAM* gene (NM_001278116.2) was detected, which was maternally inherited and validated by Sanger sequencing (Fig. 1c). The variant was also confirmed in the first abnormal fetus with corpus callosum agenesis. This mutation has been reported previously in one family with X-linked complicated spastic paraplegia, MASA syndrome, and HSAS [20]. However, there was no further functional analysis. The *L1CAM* gene variant Gly370 in our study lies in the Ig4 domain (Fig. 1d). The *L1CAM* gene variant (c.1108G > A, p.G370R) was predicted to be deleterious, according to PROVEAN, PolyPhen-2, CADD, etc. Thus, we performed a series of in vitro assays to evaluate the effects of this variant.

## Materials and Methods

### Subcloning of L1CAM cDNA and Mutagenesis

The construct which encoded the full-length human 3774 bp *L1CAM* cDNA (NM_001278116.2) with His-tag at the C-terminus and EGFP was subcloned into the pRP[Exp]-EGFP-EF1A > h*L1CAM*/6xHis vector (VectorBuilder, China). In addition, the full-length *L1CAM* cDNA with His-tag, but no EGFP, was subcloned into the pRP[Exp]-EF1A > h*L1CAM*/6xHis (VectorBuilder, China). The two constructs containing wild-type *L1CAM* cDNA were used as templates to generate the *L1CAM* cDNA variant. Replacing Gly-370 with Arg of *L1CAM* cDNA was performed by polymerase chain reaction (PCR) site-directed mutagenesis using gold medal Mix (#TSE101, Tsingke Biotechnology, China). L1-G370R variant was generated with the following primer sequence: Forward-5′-CTGGAGAATCAACAGGATCCCTGTGGAG-3′ and Reverse-5′-CTCCACAGGGATCCTGTTGATTCTCCAG-3′. The PCR product was digested using the DpnI restriction enzyme (#ER1702, Thermo Scientific, USA) and then added into DH5α (#CB101, Tiangen, China) to obtain a positive clone. The constructs of the wild-type(L1-WT) and p.G370R(L1-G370R) of *L1CAM* cDNA were verified by Sanger sequencing.

### Cell Culture and Transfection

Mouse motor neuron (NSC-34) and African green monkey kidney fibroblast-like (COS-7) cells were purchased from Tongpai (Shanghai) Biotechnology Co., Ltd. The two cells were cultured in a high glucose Dulbecco’s Modified Eagle’s Medium (#D5796, Sigma-Aldrich, USA)/DMEM (#11,965,092, Gibco, USA) supplemented with 10% fetal bovine serum (#10,099,141, Gibco, USA) and 1% streptomycin/penicillin (#C0222, Beyotime Biotechnology, China) in an atmosphere of 5% CO_2_ at 37 °C. NSC-34/COS-7 cells were transfected with plasmids, using Lipofectamine™ 2000 transfection reagent (#11,668,019, Invitrogen, USA) in accordance with standard protocols. Briefly, NSC-34/COS-7 cells were plated at a density of 3 × 10^5^ cells/9.6 cm^2^ dish, and NSC-34 cells were seeded at a density of 1.5 × 105 cells/35 mm onto poly-d-lysine-coated dishes. Twelve h later, 3 µg of plasmid DNA was added into 150 µL Opti-MEM (#31,985,070, Gibco, USA), and 9 µL of Lipofectamine™ 2000 was added to 150 µL Opti-MEM for each 6-well cell plate. For the 35-mm confocal plate, the plasmid DNA and Lipofectamine™ 2000 needed to be halved. After 5-min incubation at room temperature (RT), diluted DAN was added to diluted Lipofectamine™ 2000 reagent for another 5-min incubation. Then, cells were cultivated with DNA-lipid complex for 6 h, followed by the complete medium. Further incubation for 24 h, cells were processed for immunofluorescence and Western blotting.

### Immunofluorescence Staining

The cells were fixed with 4% paraformaldehyde for 10 min, permeabilized for 15 min in 0.3% Triton X-100, and then blocked with 5% bovine serum albumin and 0.3% Triton X-100 in phosphate-buffered saline (PBS) for 1 h at RT. The samples were incubated with mouse monoclonal anti-6XHis-tag antibody (1:200, #ab18184, Abcam, UK) and rabbit monoclonal anti-KDEL antibody (1:200, #GXP282080, GenXspan, USA) at 4 °C overnight. After being washed with PBS, cells were subjected to fluorescent secondary antibodies for 1 h at RT, including Cy3-labeled Goat Anti-Mouse IgG (H + L) antibody (1:500, #A0521, Beyotime Biotechnology, China) and Alexa Fluor 488-labeled Goat Anti-Rabbit IgG(H + L) antibody (1:500, #A0423, Beyotime Biotechnology, China). Next, cells were stained with 4′,6-diamidino-2-phenylindole (DAPI, 1:5000, #C1002, Beyotime Biotechnology, China). Finally, after being washed, the samples were imaged immediately on an FV-1000 confocal microscope (Olympus, Japan).

### Western Blotting

The cells were lysed using a RIPA lysis buffer (#P0013B, Beyotime Biotechnology, China) and a cocktail of protease inhibitors (#P1005, Beyotime Biotechnology, China) on ice for 30 min. The protein concentration was measured by a BCA Protein Assay Kit (#P0012S, Beyotime Biotechnology, China). Then, the protein extracts were separated on 8% sodium dodecyl sulfate–polyacrylamide gel electrophoresis (SDS-PAGE) and removed to nitrocellulose membranes. Next, the membranes were blocked in 5% nonfat dried milk in Tris-buffered saline containing 0.1% Tween-20 (TBST) at RT for 1 h, followed by incubation with the mouse monoclonal anti-*L1CAM* antibody (1:1000, #ab24345, Abcam, UK), rabbit monoclonal anti-phosphorylated eukaryotic initiation factor 2 antibody (*pEIF2*α, 1:1000, #3398, CST, USA), and rabbit monoclonal anti-*HSP90* antibody (1:1500, #AF1378, Beyotime Biotechnology, China), overnight at 4 °C. After being washed with TBST, the membranes were incubated with HRP-conjugated Affinipure Goat Anti-Mouse IgG (H + L) (1:5000, #SA00001-1, Proteintech, China) or HRP-conjugated Affinipure Goat Anti-Rabbit IgG (H + L) (1:5000, #SA00001-2, Proteintech, China) for 1 h at RT. After being rewashed in TBST, the protein bands were analyzed using an ECL detection system (#P0018S, Beyotime Biotechnology, China), detected using the ChemiDoc™ MP Imaging System (Bio-Rad Laboratories Inc.) and quantified using ImageJ software (V1.8.0_112).

### Deglycosylation Assay, Endoplasmic Reticulum (ER) Stress Induction, and MG132 Assay

For deglycosylation of proteins, cell lysates of the NSC-34 and COS-7 cells were treated with endoglycosidase H (Endo H) according to the manufacturer’s instructions (#P0702S, New England Biolabs, USA). After transfection for 24 h, 60 µg of total protein was denatured in glycoprotein denaturing buffer at 100 °C for 10 min. Furthermore, GlycoBuffer 3 and 0.2 µL Endo H were added to the above reaction products and incubated for 1 h at 37 °C.

For ER stress induction, cells were exposed to 0.5 µM, 1 µM, 2 µM, and 3 µM thapsigargin for 4 h (#B6614, APExBIO, US).

A medium containing 0.2 µM or 1 µM MG132 (#T510313-0001, Sangon Biotech, China) was added to the NSC-34 cells to inhibit proteasomal degradation. Furthermore, the cells were incubated for 20 h at 37 °C. In parallel, the control samples were subjected to DMSO, which is the solvent of MG132.

### Cell Aggregation Assay

NSC-34 cells were rinsed twice in PBS, detached with trypsin/EDTA, and mechanically triturated to a single cell suspension. Dissociated 1.5 × 10^5^ cells were inoculated into 24-well plates, incubated at 37 °C on a rotary shaker at 60 rpm. After a 90-min rotation, cell suspension was fixed with an equal volume of 4% paraformaldehyde, centrifuged at 1000 × *g* for 5 min, and then gently resuspended in PBS and transferred onto a microscope slide. Control cultures were fixed before rotation. Fifteen images for each condition were visualized using an inverted bright field microscope (Olympus BX60, Japan) at 200 × magnification.

### Screening of L1CAM Gene Variants in Isolated Cases with Prenatally Suspected of Corpus Callosum Agenesis Accompanied with Hydrocephalus

The 35 fetal genomic DNA samples, extracted from the amniotic fluid or fetal muscle between March 2016 and January 2020, were obtained from the Prenatal Diagnosis Center of West China Second University Hospital, Sichuan University (Chengdu, China). All cases shared corpus callosum agenesis accompanied with hydrocephalus and presented other phenotypic findings, including narrowness of brain parenchyma, brain hemorrhage, vermis of cerebellum agenesis, or the absence of cavum septi pellucidi. All DNA samples were previously detected by chromosome microarray analysis with CytoScan 750 K Array (Thermo Fisher Scientific, USA) and were found negative for clinically significant chromosomal abnormalities. All 29 exons and intron/exon boundaries of the *L1CAM* gene were sequenced by Sanger sequencing on an ABI 3500 Analyzer (Thermo Fisher Scientific, USA) to determine the *L1CAM* gene variants in one fetus. The sequences of the 12 pairs of primers were designed using Prime Primer 5 and were listed in Table 1. All sequencing results were compared with the *L1CAM* gene sequences (https://www.ncbi.nlm.nih.gov/nuccore/NC_000023.11?report=genbank&from=153861514&to=153886173&strand=true, https://www.ncbi.nlm.nih.gov/nuccore/NM_001278116.2) using the online NCBI Nucleotide BLAST (https://blast.ncbi.nlm.nih.gov/Blast.cgi). When one *L1CAM* gene variant was detected in the fetus, the *L1CAM* gene site of the parents was further validated using primers (Supplementary Table 1) designed to cover variant identified by Sanger sequencing. The pathogenicity of the *L1CAM* gene variant was evaluated according to the guidelines of the American Society of Medical Genetics and Genomics (ACMG, 2015).

**Table 1:** Primers used for the amplification and sequencing of *L1CAM* cDNA

Exon	Primer (5′-3′)	Product Size (bp)
1	Forward: ACAGCCGCTGCTGCCGCAG	352
	Reverse: CCCCGCAGGTTACCCCTCACC	
2	Forward: GGGCTTACCCAGATGTTAGTCACTA	353
	Reverse: GGAGAAGGTGAGGAGGTAGAAGATA	
3–5	Forward: CTTACTATGTCCCCTGCCATCTG	1346
	Reverse: CACAAGAAACAAGATGGGCCA	
6–11	Forward: CCCTAAGTGCTAGTCTCTGCTATGA	1903
	Reverse: GACAGACTGGGAGTTAGGAGGTAAG	
	Reverse: CAAGCAGGACGAGCGGGTGACG	
12–19	Forward: GGGGAGAGGTGACTGTCAGTTAG	2552
	Forward: GGCTGCCAATGACCAAAACA	
	Reverse: CTCCAGAGTAGCCGATAGTGACCT	
	Reverse: GCTCCCCCTGGAAATTTGGA	
20–25	Forward: AATTCGTCTTCTCTGTGTGTAGGGG	1794
	Forward: GTGAGGCGCCGGGGGCCCGCCATCA	
	Reverse: AGGAGTGACAGGGACAGGGAAAA	
	Reverse: CCCGCCTGCCTTCCATTTGTTTAG	
26–29	Forward: GTGAGGCGCCGGGGGCCCGCCATCA	2716
	Forward: GGCCCCAGACAGCTTCCCAGACAGG	
	Forward: GGGCAGACATGGTGGGGTCTCCTCA	
	Reverse: CCCGCCTGCCTTCCATTTGTTTAG	
	Reverse: GGTGCTGCCAGAGTGCGATGC	
	Reverse: AGACCAAGCACAGGCATACAGGGA	

### Statistical Analysis

Statistical analysis was performed using SPSS Statistics 24.0 (SPSS Inc., USA). Data were presented as mean ± standard deviations (SD). Differences between groups were compared using two-tailed Student’s *t*-test. *P*-values < 0.05 were considered as statistically significant.

## Results

### L1-G370R Reduced Cell Surface Expression and Induced ER Retention

**Fig. 2:**
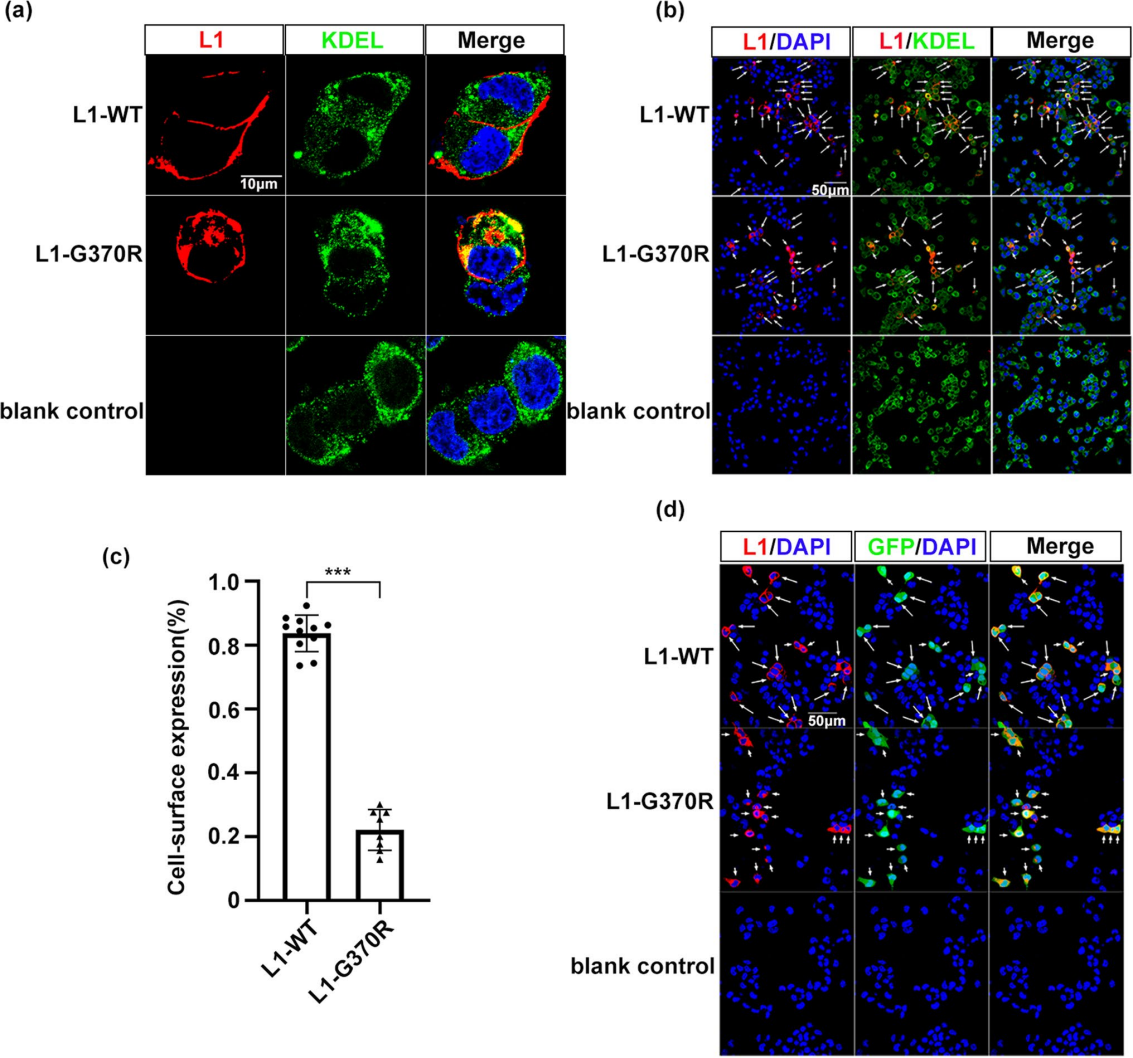
*L1CAM* p.G370R induces ER retention. **a**, **b** Cells expression of *L1CAM* proteins in NSC-34 cells transfected with L1-WT and L1-G370R constructs using antibodies to 6XHis-tag (red), which exclusively recognizes exogenous *L1CAM* protein and the ER-marker KDEL (green). Nuclei were visualized by DAPI. Arrows depict *L1CAM* located on the cell surface. Arrowheads depict *L1CAM* co-localization with ER marker. **c** Diagram showing the percentage of cell surface expression of wild-type and mutant human *L1CAM* in NSC-34 cells. L1-WT, 83.66 ± 5.74%; L1-G370R, 22.11 ± 6.40%. Data represent means ± SD; ***P < 0.001. **d** Confocal images of NSC-34 cells co-expression of human *L1CAM* and EGFP constructs. GFP-positive cells presenting *L1CAM* cell surface expression were tagged by arrows, while barely presented *L1CAM* cell surface expressions were tagged by arrowheads. Scale: 10 μm (**a**), 50 μm (**b**, **d**)

We performed *L1CAM* c.1108G > A mutation using PCR site-directed mutagenesis, to understand the pathogenic nature of *L1CAM* variant p.G370R on NSC-34 and COS-7 cells, and the sequences were checked by Sanger sequencing. The NSC-34 cells transfected with L1-WT or L1-G370R were double-stained with anti-6XHis-tag antibody that specifically recognizes exogenous *L1CAM* protein and the ER-retrieval motif KDEL antibody to investigate the changes in subcellular localization. A high percentage of L1-WT was localized in the cell membrane surface (83.66 ± 5.74%). However, we found only 22.11 ± 6.40% of L1-G370R expressed on the cell surface, presenting a redistribution to ER, as tested by co-localization with the ER-marker KDEL, resulting in 73.57% of reduction of cell surface *L1CAM* protein expression (*P* < 0.001, Fig. 2a–c).

NSC-34 cells were co-transfected *L1CAM* with GFP-expressing plasmids to examine transfection efficiencies and protein expression levels of L1-G370R. There were almost no differences in transfection efficiencies between the cells transfected with L1-WT or L1-G370R. Furthermore, all GFP-positive cells expressed *L1CAM* protein on the cell surface or redistributed in intracellular sites (Fig. 2d). This observation indicated that the differences in cell surface expression have not resulted from transfection efficiencies or *L1CAM* expression levels.

### Posttranslational Modification Was Impaired for L1-G370R

**Fig. 3:**
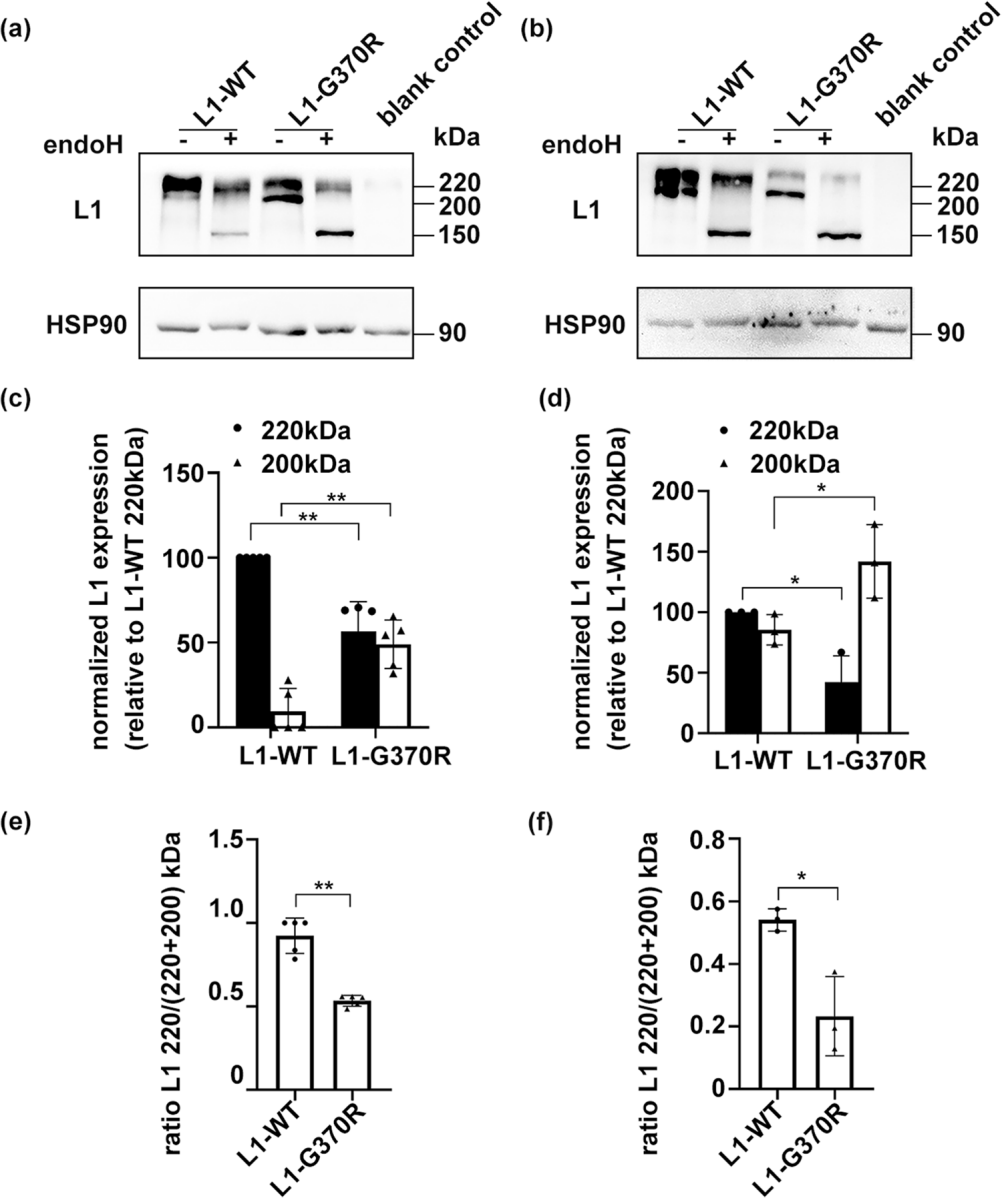
Cell lysates from NSC-34 to COS-7 cells expressing wild-type (L1-WT) or mutant L1 (L1-G370R) were treated with endoglycosidase H (Endo H). **a**, **b** *L1CAM* immunoblot analysis showing Endo H specifically cleaved high-mannose type of *L1CAM* (200 kDa), whereas it had no work to complex-mannose type (220 kDa). **c**, **d** Histogram showing the 220-kDa form of L1-G370R was reduced, while the 200-kDa form was increased, compared to L1-WT. **e**, **f** Histogram showing the 220/(220 + 200) kDa ratio of L1-G370R was reduced relative to L1-WT. NSC-34 cells, **a**, **c**, **e**; COS-7 cells, **b**, **d**, **f**

Cell lysates of transfected NSC-34 and COS-7 cells were subjected to SDS–PAGE and immunoblotting with anti-*L1CAM* antibody, revealing two bands identified at 220 and 200 kDa (Fig. 3a, b). The 220-kDa band corresponds to a full-length complex-mannose type of *L1CAM* located at the cell surface, generated through posttranslational modification in the Golgi apparatus. On the other hand, the 200-kDa band might represent an incompletely processed high-mannose type of *L1CAM*, which is generated only through co-translational modification in the ER [21, 22]. We found that in the NSC-34 and COS-7 cells, 220-kDa form, as well as the 220/(220 + 200) kDa ratio of L1-G370R, reduced, while the 200-kDa form increased, compared to L1-WT (*P* < 0.05, Fig. 3c–f). The cell lysates were digested with Endo H, which exclusively decomposes high-mannose oligosaccharides, but it is useless to decompose the complex-mannose oligosaccharides [23]. After incubation with Endo H, the 200-kDa form of L1-WT or L1-G370R was shifted to a 150-kDa fragment of *L1CAM*. In contrast, Endo H showed no significant effects on the 220-kDa band, representing a mature form of *L1CAM* (Fig. 3a, b). These findings demonstrated that the *L1CAM* 200-kDa form was likely to accumulate in the ER and was not transported through the Golgi organelle to the cell surface.

### L1-G370R Did Not Induce ER Stress

**Fig. 4:**
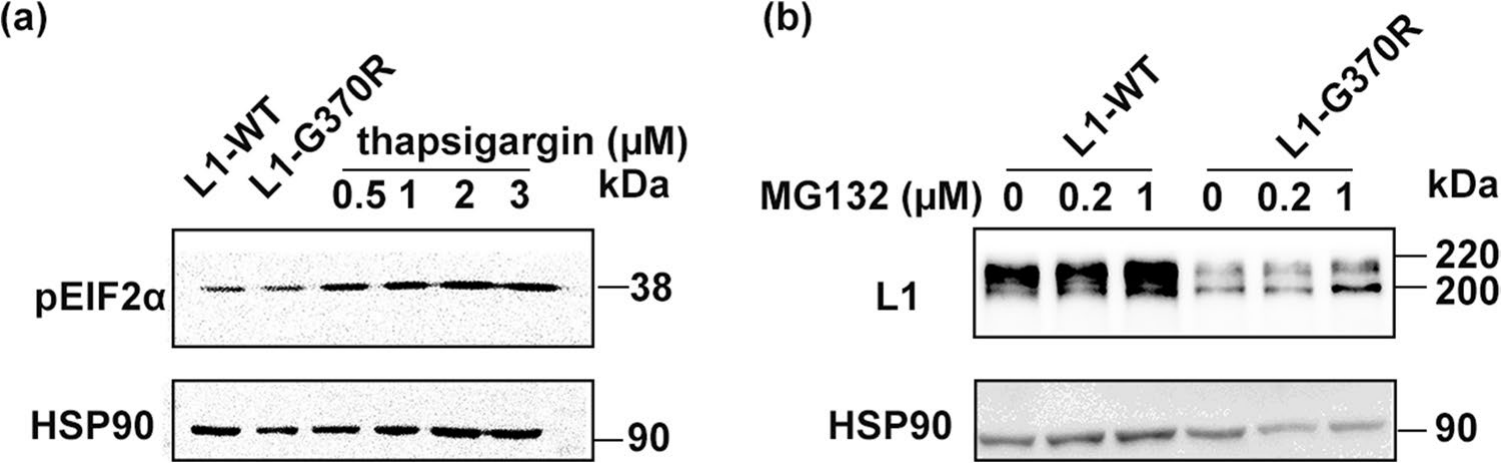
L1-G370R does not induce ER stress. **a** *pEIF2*α expression levels from NSC-34 expressing L1-G370R were almost not raised compared with cells transfected with L1-WT. As a positive control, cultures were subjected to 0.5 µM, 1 µM, 2 µM, and 3 µM thapsigargin for 4 h. **b** The expression levels of L1-WT and L1-G370R were gradually upgraded after treatment with 0.2 µM and 1 µM proteasome inhibitor MG132 for 20 h

Cells initiate the ER-associated degradation (ERAD) to avoid the excessive deposition of unfolded or misfolded proteins in the ER via the ubiquitin–proteasome pathway. If the load of the ER exceeds its capacity, cells may go into ER stress leading to cell death. To investigate whether ER dislocation of L1-G370R induces ER stress, we detected the *pEIF2*α level, the ERAD downstream molecule. There were almost no upgraded levels of *pEIF2*α in NSC-34 cells following overexpression of L1-G370R compared with the cells transfected with L1-WT, whereas treatment with the ER stress inducer thapsigargin increased the *pEIF2*α (Fig. 4a). When transfected NSC-34 cells were co-cultured with 0.2 µM or 1 µM proteasome inhibitor MG132 for 20 h, the expressing levels of L1-WT and L1-G370R improved, indicating L1-WT and L1-G370R were degraded by the ERAD (Fig. 4b). The collected data suggest impaired cell surface expression, rather than cytotoxicity caused by ER retained L1-G370R, is the key pathogenesis motif for the L1 syndrome.

### L1-G370R Impaired L1CAM-Dependent Cell–Cell Adhesion

**Fig. 5:**
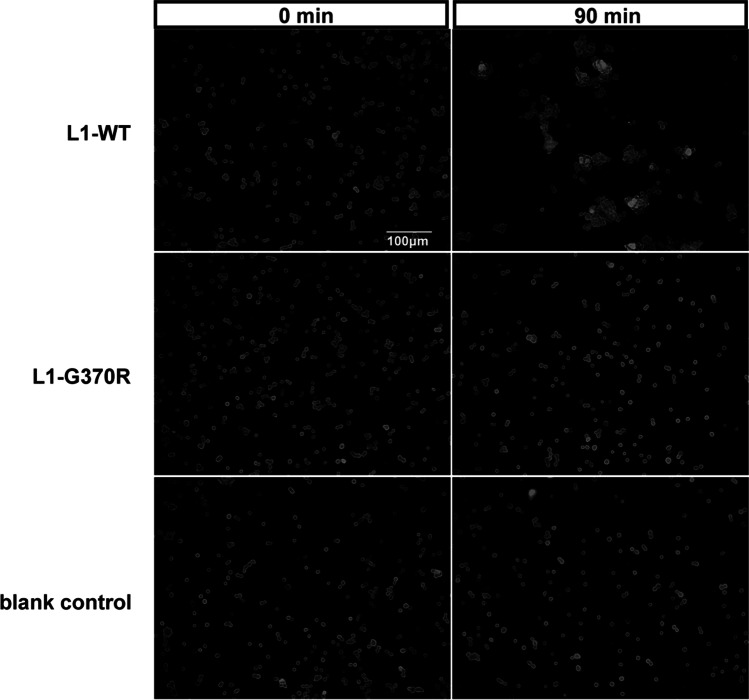
L1-G370R disrupted *L1CAM*-dependent cell–cell adhesion NSC-34 cells expressing L1-WT induced cell aggregation, but cells expressing L1-G370R and blank control almost failed to form cell aggregates. Scales: 100 μm

*L1CAM* can promote cell–cell binding by homophilic adhesion in vitro [24]. Binding assays involving L1-WT and L1-G370R protein expressed on transfected NSC-34 cells were applied to analyze cell–cell binding ability. After the separation of transfected cells, they were given a chance to form aggregates for 90 min. Aggregation was completed within 90 min in NSC-34 cells transfected with the L1-WT construct. In contrast, cells expressing L1-G370R or blank control almost failed to form cell aggregates (Fig. 5). Thereby, *L1CAM*-mediated cell aggregation was disrupted by p.G370R. The collected data suggest *L1CAM* variant p.G370R was likely related to L1 syndrome (Table 2).

**Table 2:** Summary of the results of functional experiments

Nucleotide change	Exon	Amino acid change	Protein domain affected		Combinatorial prediction
c.1108G > A	9	p.G370R	Ig4		Affects function
Protein maturation	Surface expression	Posttranslational modification	ER stress	Cell–cell adhesion	
Incomplete	Reduced	Impaired	Uneffected	Impaired	

### Screening for L1CAM Gene Mutations

**Fig. 6:**
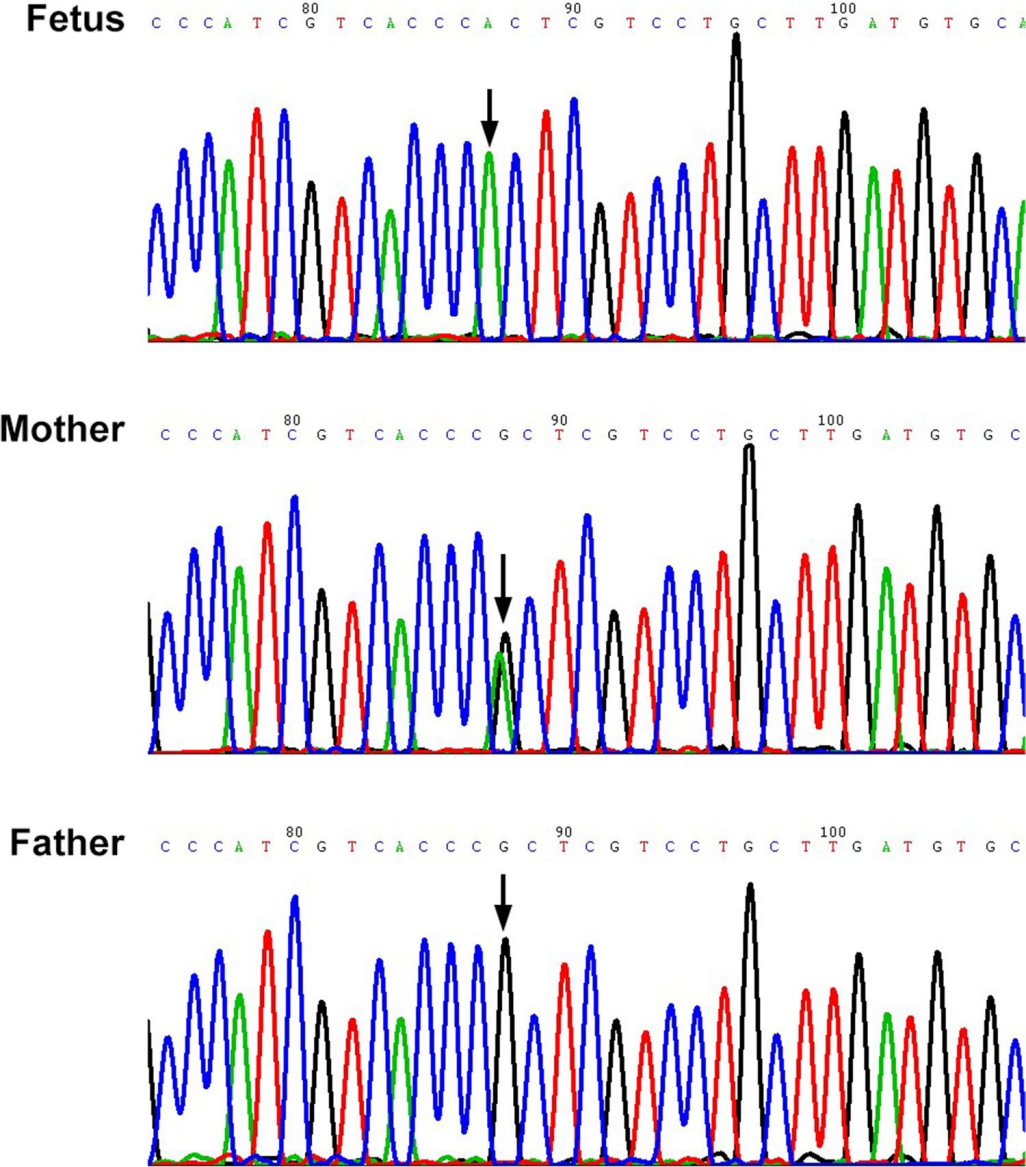
Sanger sequences of the *L1CAM* gene mutation (c.550C > T). Hemizygous mutation detected in the fetus presented corpus callosum agenesis accompanied with hydrocephalus. The heterozygous mutation detected in the fetus’ mother but not in the fetus’ father. Mutation was indicated by arrow

In families suspected of L1 syndrome, the determination of *L1CAM* gene mutations would give a chance of prenatal diagnosis, ultimately avoiding the congenital disabilities relevant to *L1CAM* gene mutation. In the prenatal, ultrasound attention should be paid to hydrocephalus and agenesis of the corpus callosum related to L1 syndrome. We used Sanger sequencing to detect *L1CAM* gene variants in 35 fetal genomic DNA samples. They all shared corpus callosum agenesis accompanied with hydrocephalus. After blasting sequencing results to *L1CAM* gene sequences (NC_000023.11), one single missense nucleotide variation of *L1CAM* gene (c.550C > T, p.R184W) was detected in one fetus and validated by Sanger sequencing (Fig. 6). Through clinical data, published literature, and bioinformatic analysis, the *L1CAM* gene single-nucleotide variant (p.R184W) is a likely pathogenic mutation [21, 22, 25, 26].

By only detecting 29 exons and intron/exon boundaries using Sanger sequencing, our data indicated that the detection rate of *L1CAM* gene mutant was 3% (1/35) in isolated fetuses prenatally suspected of corpus callosum agenesis accompanied with hydrocephalus.

## Discussion

Here, a *L1CAM* gene exonic missense variant (c.1108G > A, p.G370R) was identified in two induced fetuses (abnormal fetuses), who presented corpus callosum agenesis accompanied with hydrocephalus. Clinical data, published literature, online database, and online tools suggest the *L1CAM* gene single-nucleotide variant is a likely pathogenic mutation. In vitro assays indicated that this variant might relate to L1 syndrome. The *L1CAM* gene exonic missense variant (c.1108G > A, p.G370R) reduced cell surface expression, induced partial ER accumulation, affected posttranslational processing, and, finally, impaired the *L1CAM* homophilic adhesive ability and, thus, loss of function. Thirty-five isolated cases prenatally suspected of corpus callosum agenesis accompanied with hydrocephalus were screened to acquire detection rate of *L1CAM* gene variant. Consequently, we found one *L1CAM* gene single missense nucleotide variation (c.550C > T, p.R184W) in one fetus. Our data indicated that the detection rate of *L1CAM* gene mutant was 3% (1/35) in isolated fetuses prenatally suspected of corpus callosum agenesis accompanied with hydrocephalus, using Sanger sequencing to detect 29 exons and intron/exon boundaries. Taken together, our results provided evidence that the *L1CAM* gene missense variant (c.1108G > A, p.G370R) may relate to L1 syndrome. The findings of this study suggest a potential possibility of *L1CAM* gene screening for prenatal diagnoses for fetuses presented corpus callosum agenesis accompanied with hydrocephalus.

Previous studies have shown that Ig1-Ig4 domains of *L1CAM* compose a horseshoe conformation that mediates homophilic adhesion function [27, 28]. The *L1CAM* gene variant Gly370 in our study lies in the Ig4 domain, a key amino acid domain. Fransen et al. suggested that extracellular missense mutations affecting key domains of amino acids might cause a more devastating phenotype than those affecting surface sites [29]. Also, Carlos Ruiz et al. reported the G370R mutation in a family with X-linked complicated spastic paraplegia, MASA syndrome, and HSAS [20]. Although it is tempting to consider this *L1CAM* gene missense variant (c.1108G > A, p.G370R) as pathogenic, it is particularly important to further identify the functional effects before drawing any firm conclusions. Therefore, we performed this study.

When NSC-34 cells were transiently transfected with L1-G370R plasmids, significantly reducing the amounts of cell surface protein and inducing redistribution to ER that co-immunostained to KDEL were observed in most transfected cells, compared with L1-WT. Confocal images of NSC-34 cells transfected with L1CAM wild type or mutant with GFP-expressing plasmids revealed similar transfection efficiencies and protein expression levels for the two constructs. These results revealed that the differences of cell surface expression of L1-G370R were not caused by transfection efficiencies or protein expression levels. Bateman et al. have predicted that *L1CAM* gene variant p.G370R might alter the conformation of this protein [30]. Accumulation in ER of misfolded transmembrane proteins has been shown to be pathological for several genetic disorders, for example, cystic fibrosis and Kallmann syndrome [31, 32]. Therefore, the ER accumulation of misfolded L1-G370R might be pathogenic.

The glycoprotein *L1CAM* passes through ER co-translational processing, Golgi complex posttranslational processing, and is finally transferred to the cell surface [33]. We thus studied whether ER retainability of L1-G370R may be immature protein. By immunoblotting, we found that the 220-kDa form and the 220/(220 + 200) kDa ratio of L1-G370R were reduced, but the 200-kDa form increased compared to L1-WT. Also, when treated with Endo H, the molecular mass of 200-kDa form of L1-WT or L1-G370R was shifted to 150-kDa. These findings indicated that the 200-kDa form of L1-G370R accumulated in the ER did not complete Golgi apparatus posttranslational modification, thus impairing the cell surface 220-kDa *L1CAM* corresponding to the mature form. Moulding et al. have demonstrated that the R184Q and D598N *L1CAM* proteins impair cell surface expression and incomplete posttranslational processing in astrocytes, Vero, COS-7, and CHO cells [21]. Furthermore, the *L1CAM* gene missense mutation (p.W635C) accumulated in the ER also interferes with posttranslational modification [34]. These results align with our results that p.G370R causes defects in the trafficking of *L1CAM* to the cell surface.

In the process of normal protein synthesis, unfolded or misfolded proteins are often produced. Cells initiate ERAD to remove unfolded or misfolded proteins that are retained in the ER by cytosolic proteosomes. If this process fails, it may induce ER stress that may render cells to death. Several neurological disorders are caused by ER stress resulting from ER accumulation of mutated protein [35, 36]. However, our study indicated that NSC-34 cells overexpressing L1-G370R did not induce ER stress. This may be because mutant *L1CAM* protein is efficiently degraded by ERAD [37]. Consistent with our hypothesis, our experiment revealed that L1-WT and L1-G370R proteins were upgraded when proteasomal degradation was inhibited. In line with our data, the *L1CAM* gene variants R184Q and W1036L have been identified as two pathogenic mutations but do not induce ER stress in NSC-34 cells [22]. These findings demonstrated that impaired cell surface expression rather than cytotoxicity caused by ER retained L1-G370R is the critical pathogenesis for the L1 syndrome.

Earlier studies concluded that defects in *L1CAM* homophilic binding are likely to contribute to L1 syndrome [25]. Reducing cell surface expression would impair *L1CAM* mediate homophilic binding and signaling in cells; thus we performed a cell aggregation assay. Compared to NSC-34 cells overexpressing L1-WT, *L1CAM*-dependent cell–cell adhesion was impaired in NSC-34 cells overexpressing L1-G370R, which agrees with the research showing that L1-G370R dramatically reduces ligand binding through fluorescent microspheres assay [25]. Also, Mariola et al. have shown that *L1CAM* gene variants p.W635C and p.V768I affect *L1CAM*-dependent homophilic binding [34, 38].

Congenital corpus callosum agenesis accompanied with hydrocephalus is a rare and exceedingly heterogeneous condition that can result from multiple causes. It can be detected by antenatal ultrasound but poses a great challenge to prenatal diagnosis as the outcome is variable. An online search for “corpus callosum agenesis and hydrocephalus” on Online Mendelian Inheritance in Man (OMIM) resulted in 93 entries. Some condition of corpus callosum agenesis accompanied with hydrocephalus has recognizable syndromes, including L1 syndrome (MIM #304,100), Chudley-McCullough syndrome (MIM #604,213), agenesis of corpus callosum, cardiac, ocular, and genital syndrome (MIM #618,929), MASA syndrome (MIM #303,350), and so on. However, some individuals with corpus callosum agenesis accompanied with hydrocephalus do not have a clearly inherited cause [39, 40]. The identifiable genes for the above identifiable syndromes, respectively, are *L1CAM*, G-protein signaling modulator (*GPSM2*), and cadherin (*CDH2*). We screened 35 unrelated fetuses prenatally suspected of corpus callosum agenesis accompanied with hydrocephalus for *L1CAM* gene variants to acquire a detection rate of *L1CAM* gene variant in these cases. Consequently, we found one single missense nucleotide variation *L1CAM* gene (c.550C > T, p.R184W) in one fetus. The L1CAM gene variant (c.550C > T, p.R184W) was predicted to be deleterious, according to PROVEAN, PolyPhen-2, MUpro, CADD, etc. Furthermore, the amino acid change at this site (R184) was pathogenic which have been published by Schäfer et al. [21, 22, 25, 26]. Our data indicated that the detection rate of L1*CAM* gene mutant was 3% (1/35) in isolated fetuses prenatally suspected of corpus callosum agenesis accompanied with hydrocephalus, using Sanger sequencing to detect 29 exons and intron/exon boundaries. A previous report indicated that approximately 5% of children with congenital hydrocephalus was X-chromosomal hydrocephalus caused by *L1CAM* gene mutations [41]. Another report indicated that the detection rate of *L1CAM* gene mutant was 16% in X-chromosomal hydrocephalus without a family history [39, 40]. Due to low incidence of this condition, detection method application, and selection bias in the reported study, the actual prevalence is difficult to estimate. In the future, we will further increase the sample size and apply other genetic methods for search. *L1CAM* gene mutation detection can be offered to fetuses presented corpus callosum agenesis accompanied with hydrocephalus.

In conclusion, we provided two induced fetuses (abnormal fetuses) suspected of L1 syndrome with *L1CAM* gene variant (c.1108G > A, p.G370R). Clinical data, published literature, online database, and online tools suggest the *L1CAM* gene single-nucleotide variant is a likely pathogenic mutation. In addition, in vitro analyses indicated that the *L1CAM* gene mutation (c.1108G > A, p.G370R) reduced cell surface expression, induced partial ER retention, affected posttranslational modification, reduced protein’s homophilic adhesive ability, but did not induce ER stress, which was likely to be associated with the L1 syndrome. The molecular mechanisms involving in L1 syndrome should be further studied. Ultimately, we screened 35 unrelated fetuses prenatally suspected of corpus callosum agenesis accompanied with hydrocephalus for *L1CAM* gene variants by Sanger sequencing. Consequently, one *L1CAM* gene single missense variant (c.550C > T, p.R184W) was detected in one fetus. Our results provide evidence that the *L1CAM* gene missense variant (c.1108G > A, p.G370R) may relate to L1 syndrome. The findings of this study suggest a potential possibility of *L1CAM* gene screening for prenatal diagnoses for fetuses presented corpus callosum agenesis accompanied with hydrocephalus.

## Supplementary Information

Below is the link to the electronic supplementary material.

Supplementary file1 (DOCX 14 KB)

## Data Availability

Not applicable.
