# Supplementary material for: L1 Syndrome Prenatal Diagnosis Supplemented by Functional Analysis of One L1CAM Gene Missense Variant
Source: Reprod Sci. 2021 Dec 16;29(3):768–80. doi: 10.1007/s43032-021-00828-4 (PMC8863719; doi:10.1007/s43032-021-00828-4)
Supplement: Supplementary file 1 — Supplementary file1 (DOCX 14 KB) [file 43032_2021_828_MOESM1_ESM.docx]

| **Primers** | **Forward** | **Reversed** |
| --- | --- | --- |
| c.1108G>A | TTCCTCACCCTCCTCTTCCT | TCACCTACCAGAACCACAACA |
| c.550C>T | CCTTCTGAATGATGGTCCT | CGGATCTACTGGATGAACA |
